# Mimicking hypomethylation of FUS requires liquid–liquid phase separation to induce synaptic dysfunctions

**DOI:** 10.1186/s40478-023-01703-w

**Published:** 2023-12-18

**Authors:** Seung Chan Kim, Scott J. Mitchell, Seema Qamar, Daniel J. Whitcomb, Marc-David Ruepp, Peter St George-Hyslop, Kwangwook Cho

**Affiliations:** 1 Department of Basic and Clinical Neuroscience, Institute of Psychiatry, Psychology and Neuroscience, UK-Dementia Research Institute, Maurice Wohl Clinical Neuroscience Institute, King’s College London, London, SE5 9NU UK; 2 Department of Clinical Neurosciences, Cambridge Institute for Medical Research, University of Cambridge, Cambridge, CB2 0XY UK; 3 Bristol Medical School, University of Bristol, Bristol, BS1 3NY UK; 4 Department of Medicine (Division of Neurology), University Health Network and Tanz Centre for Research In Neurodegenerative Diseases, Temerty Faculty of Medicine, University of Toronto, Toronto, ON M5S 3H2 Canada; 5 Taub Institute For Research On Alzheimer’s Disease and the Aging Brain, Department of Neurology, Columbia University Irving Medical Center, 630 West 168 Street, New York, NY 10032 USA

## Abstract

The hypomethylation of fused in sarcoma (FUS) in frontotemporal lobar degeneration promotes the formation of irreversible condensates of FUS. However, the mechanisms by which these hypomethylated FUS condensates cause neuronal dysfunction are unknown. Here we report that expression of FUS constructs mimicking hypomethylated FUS causes aberrant dendritic FUS condensates in CA1 neurons. These hypomethylated FUS condensates exhibit spontaneous, and activity induced movement within the dendrite. They impair excitatory synaptic transmission, postsynaptic density-95 expression, and dendritic spine plasticity. These neurophysiological defects are dependent upon both the dendritic localisation of the condensates, and their ability to undergo liquid–liquid phase separation. These results indicate that the irreversible liquid–liquid phase separation is a key component of hypomethylated FUS pathophysiology in sporadic FTLD, and this can cause synapse dysfunction in sporadic FTLD.

**Supplementary Information:**

The online version contains supplementary material available at 10.1186/s40478-023-01703-w.

## Introduction

Fused in sarcoma (FUS) is a DNA/RNA-binding protein in which mutations and altered post-translation modifications (especially hypomethylation of arginine residues) give rise to pathological condensates that cause FUS-associated frontotemporal lobar degeneration (FTLD-FUS) and familial amyotrophic lateral sclerosis (fALS-FUS) [1–3]. Under physiological conditions FUS is mainly located in the nucleus, from where it shuttles to the cytoplasm to perform roles at dendritic and axonal compartments. A key feature of FUS, essential for its function, is its ability to undergo liquid–liquid phase separation (LLPS) to form physiologically reversible biomolecular condensates (hereafter “condensates”). These condensates are thought to underpin the role of FUS ribonucleoprotein granules in supporting regulated, specialised protein synthesis in distal neuronal compartments [2–4]. The formation of these condensates is normally a reversible process. However, dysregulation of this process is a common feature across FUS-related pathological conditions. In these conditions, the pathological FUS species form stable fibrillar inclusions [1–3]. These pathological condensates are typically mislocalised to the cytoplasm of spinal and hippocampal neurons of ALS and FTLD patients [5–7], and are characteristic neuropathological features of fALS-FUS and FTLD-FUS. These aberrant cytoplasmic condensates are thought to play a central role in spinal and hippocampal synaptic dysfunction in these disorders [10, 11]. However, two distinct biophysical mechanisms accelerate the formation of these pathological fibrillar condensates. In fALS-FUS the increased propensity of FUS to form irreversible condensates is largely driven by the presence of missense mutations. In contrast, in FTLD-FUS the pathological condensation is driven by hypomethylation of arginine residues in FUS [8, 9]. These differences in how the pathological condensates are formed raises the possibility that there may also be distinctions in the molecular mechanism(s) by which they induce neuronal dysfunction. To date, much of the research exploring the mechanism of FUS pathology in neurodegeneration has focused on the missense mutations associated with fALS-FUS [10]. Much less is currently known about the molecular/cellular pathobiology of hypomethylated FUS associated with sporadic FTLD-FUS [9].

In FTLD-FUS, which accounts for approximately 10% of all FTLD cases, FUS inclusions have been observed in multiple brain regions, including the hippocampal pyramidal layer [11, 12]. Thereby, indicating that FUS inclusions may contribute to the cognitive deficits observed during FTLD. Previous studies have suggested a role for FUS in synapse regulation and have demonstrated interactions with key synapse associated proteins (e.g., PSD95, GluA1) [13–16]. Recently, knock-out of FUS in CA1 hippocampal pyramidal cells was shown to alter excitatory synaptic function in a region-specific manner [13]. Collectively, positioning FUS, and its dysregulation as a potential driver of FTLD associated pathophysiology.

Biochemically, in FTLD-FUS, FUS hypomethylation accelerates pathological condensation by increasing the strength of inter- and intra-molecular arginine: tyrosine cation-pi interactions between protons in the guanidine moiety in the arginine side chains and electrons in the aromatic rings in the tyrosine side chains in FUS [2, 17]. However, the enzymatic basis of this arginine hypomethylation is unclear. As a result, suitable models have not been available to investigate how hypomethylated FUS condensates cause neuronal dysfunction. Specifically, it is unknown whether the neuronal dysfunction arises from the dysregulated localisation or the heightened LLPS ability of hypomethylated FUS, or both.

Previously we have shown that the biophysical effects of FUS hypomethylation can be discretely modelled by increasing the number of arginine residues (by 9,16 or 21 extra arginines) in the poorly conserved, intrinsically disordered, low complexity domain (LCD) of FUS [2]. These constructs produce properly folded FUS proteins whose CD spectra are indistinguishable from wild type methylated FUS or wild type hypomethylated FUS [2]. However, they display an arginine-dose-dependent increase in the cation-pi drive and accelerated formation of irreversible fibrillar FUS condensates upon ageing or upon expression in cells. The resulting pathological FUS condensates exhibit aberrant biophysical and functional properties similar to those of hypomethylated FUS purified from human FTLD-FUS brain and from Adenosine dialdehyde (AdOx)-treated cells [2, 3]. Specifically, these pathophysiological properties include binding to fluorescent dyes like pFTAA, and solubility characteristics similar to FUS condensates in FTLD-FUS [2, 3]. In the current experiments we use the FUS construct with 16 extra arginine residues (FUS-16R) because we have previously shown that FUS-16R displays properties both in recombinant protein and in cell-based experiments that most closely mimic modest degrees of arginine hypomethylation observed in FTLD-FUS brain and AdOx-treated cells [2, 3].

The conventional approach to the investigation of the pathobiology of hypomethylated FUS in FTLD-FUS has been to inhibit arginine methylation using small molecules such as AdOx (adenosine dialdehyde). AdOx is a global methyltransferase inhibitor. However, AdOx causes broad changes in one-carbon metabolism, and results in the hypomethylation of DNA and numerous other proteins in addition to demethylation of FUS. As a result, the effects of AdOx on neuronal function are likely to be much broader than just hypomethylation of FUS. As a result, the “hypomethylation-mimicking” FUS constructs (especially FUS-16R) provide a discrete, powerful new tool that circumvents the limitations of AdOx. Specifically, it allows investigation of the effects of increased cation-pi driven condensation of FUS arising from FUS hypomethylation.

Here we capitalise on this tool to mimic hypomethylation of FUS in live neurons to reveal how hypomethylated FUS condensates dysregulate synaptic function. To dissect if over condensation or specific properties of the FUS condensates are responsible for pathological progression, we created two modified FUS-16R constructs. One encompasses a powerful c-terminal SV40 nuclear localisation signal (FUS-16R-NLS) that retains the FUS-16R protein in the nucleus. The other impairs LLPS by mutating 27 Tyrosine to Serine substitutions in the low-complexity domain, thereby reducing the cation-pi drive and impairing LLPS (FUS-16R-LLPS) [18–21]. We then applied these constructs to investigate the dynamics and activity-induced recruitment of FUS in the CA1-Schaffer Collateral synapse circuit model of the hippocampus [2, 22]. The experiments outlined below demonstrate that stable, fibrillar condensates form in an activity-dependent fashion, and cause synaptic dysfunction. This synaptic dysfunction is dependent upon both the ability of FUS to form pathological condensates, and on the localisation of these condensates in distal neuronal compartments.

## Results

### FUS-16R induced condensates in the soma and dendrite exhibit spontaneous and activity induced movement

**Fig. 1:**
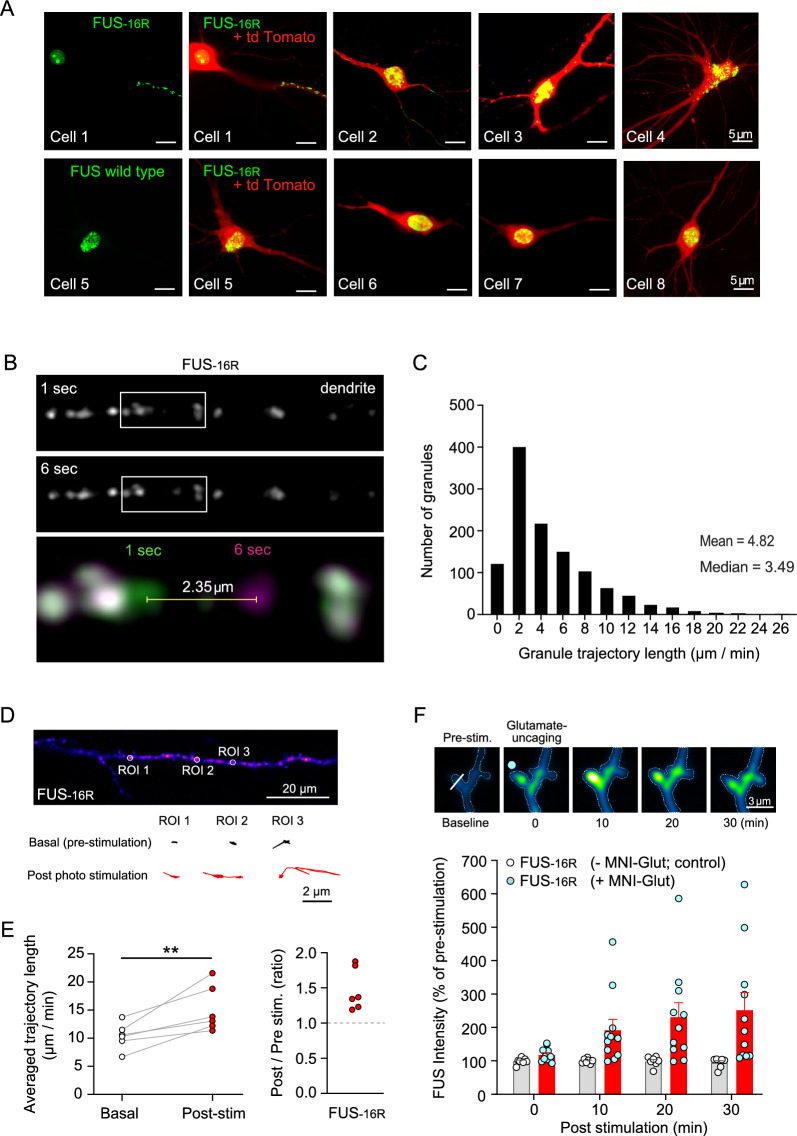
FUS-16R causes the formation of dendritic inclusions which exhibit spontaneous movement that is enhanced by neuronal activity*.* **A** Representative confocal images illustrating the somatic and dendritic region of CA1 neurons expressing either FUS-16R-EYFP (FUS-16R) and td-tomato (top panels; cell 1–4) or FUS-WT-EYFP (FUS-WT) and td-tomato (bottom panels; cell 5–8). **B** Representative straightened time-lapse image of an apical dendritic region transfected with FUS-16R top panel (1 s), middle panel (6 s). Live imaging allowed for the tracking of individual FUS-16R condensates as indicated by the pseudo-colour merged time-lapse image (bottom panel; green 1 s; magenta 6 s). **C** Quantification of the FUS-16R-EYFP granule trajectory length recorded from individual granules. **D** Representative pseudo-coloured heat map of FUS16R condensate intensity in a dendritic region of interest. Dendritic FUS16R condensates are highlighted (ROI1-3) and their movement trajectory over a 1-min period is plotted for pre-stimulation (baseline; black line) and post-stimulation (Chrismon depolarisation; red line). **E** Quantification of normalised FUS-16R-condensate movement (averaged 5–6 ROIs per cell, n = 6). Average trajectory length was longer post-stimulation (p = 0.009373, paired t-test). **F** Representative multiphoton timelapse (10-min interval) heat maps of FUS-16R intensity at a single CA1 dendritic spine prior to and following single spine glutamate uncaging. A single spine was stimulated (cyan dot) and the FUS-16R intensity was measured by line scan across spine head. Histogram (below) illustrating FUS-16R condensate signal following stimulation in the presence (red bars) and absences (grey bars) of MNI-glutamate. Stimulation in the presence of MNI-glutamate significantly increased condensate signal at 20- (p = 0.0485, post hoc Tukey) and 30-min (p = 0.0214, post hoc Tukey) post-stimulation. **p < 0.01, paired t-test

We initially compared the localisation and dynamics of tagged FUS condensates which mimic hypomethylation of FUS (FUS-16R) and wild type FUS (FUS-WT), expressed via biolistic transfection in CA1 hippocampal neurons (Fig. 1). FUS-WT is present as abundant nucleoplasmic granules, which is the predominant physiological subcellular location for wild type FUS when expressed at endogenous levels. Interestingly, in our model we did not observe any FUS-WT at synaptic locations [14–16]. In contrast, FUS-16R forms modest numbers of granules in the soma and nucleus, together with abundant granular assembles in dendrites (Fig. 1A). Within the apical dendrite, FUS-16R condensates exhibit dynamic movement and translocate to dendritic spine-like structures (Fig. 1B, C). Additionally, we illustrate that the FUS-16R condensates were not affected by incubation with 1,6 Hexanediol, a compound known to disassemble biomolecular condensates [20, 23], this suggests that FUS-16R formed condensed assemblies in the form of gels and/or fibrillar aggregates (Additional file 1: Fig. S1).

Next, we examined whether neuronal activity alters the dynamics and localisation of the FUS condensates within spines and dendrites. To test this, we utilised the red-shifted channel rhodopsin chrimson [24] to optically induce dendritic depolarisation. We found that chrimson mediated depolarisation (647 nm, 200 ms, 0.4 Hz, 5 min) increased the movement of the FUS-16R condensates in apical dendritic regions of the CA1 neuron (p = 0.009, paired t-test; n = 7 cells per group; Fig. 1D, E). To further explore this phenomenon, we next asked whether single spine activation with uncaged glutamate (MNI-Glutamate) was sufficient to recruit FUS condensates to the activated spines. We found that uncaging glutamate at the spine induced a transient increase in FUS-16R in the spine (Fig. 1F) (F_(4,28)_ = 4.732, p = 0.0048, one-way RM-ANOVA, Fig. 1F).

### Validation of NLS and LLPS FUS-16R constructs

**Fig. 2:**
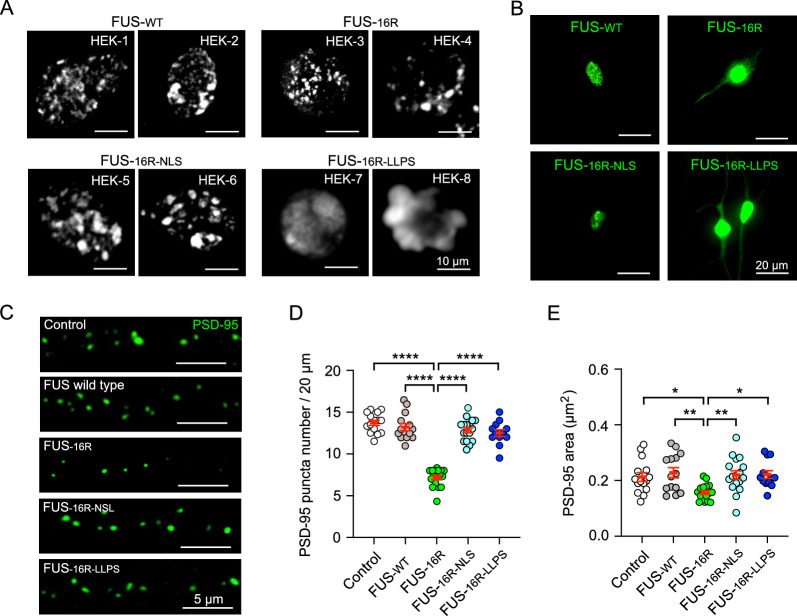
All FUS-16R constructs, except FUS-16R-LLPS, can form aggregates, and only FUS-16R induced a significant reduction in the PSD95 puncta density and area*.* **A** Representative images of FUS-WT, FUS-16R, FUS-16R-NLS and FUS-16R-LLPS transfected HEK cells, illustrating the ability of all constructs except FUS-16R-LLPS to form FUS aggregates. **B** Representative images of CA1 neurons expressing either FUS-WT, FUS-16R-EYFP (FUS-16R), FUS-16R-NLS or FUS-16R-LLPS. **C** Representative images of endogenous PSD95 puncta (green) in control, FUS-WT, FUS-16R, FUS-16R-NLS and FUS-16R-LLPS transfected neurons. **D**, **E** Analysis of PSD95 puncta number (**D**) and puncta area (**E**) for control (n = 15), FUS-WT (n = 14), FUS-16R (n = 17), FUS-16R-NLS (n = 17) and FUS-16R-LLPS (n = 12) transfected neurons within 20 µm-sized ROIs. * p < 0.05, ** p < 0.01, **** p < 0.0001, post hoc Tukey analysis (One way ANOVA)

As we have previously shown the addition of the 16 additional arginine residues to FUS induced increased cation-pi interactions and drives LLPS and subsequent fibrillar formation [2]. In the present work, we wished to investigate what properties of FUS-16R condensates could be responsible for driving pathophysiology, therefore we utilised previously validated mutations to alter key properties of FUS-16R [19–21]. Specifically, we induced the nuclear only localisation of FUS16R (FUS-16R-NLS [20]) by the addition of a c-terminal SV40 nuclear localisation signal or impaired the ability of FUS-16R to undergo LLPS (FUS-16R LLPS [19]) by the addition of 27 tyrosine to serine substitutions in the N-terminal domain (Additional file 1: Fig. S2). The various FUS constructs were expressed in HEK cells, and as expected, FUS-WT, FUS-16R, FUS-16R-NLS formed clear distinct condensates and FUS-16R-LLPS did not. These findings indicated that FUS-16R-LLPS impairs the ability of FUS-16R to undergo LLPS and forming FUS aggregates (Fig. 2A). Subsequently, we biolistically transfected the constructs into CA1 neurons and observed that FUS-WT and FUS-16R-NLS remain nuclear bound, FUS-16R forms condensates throughout the neuron and FUS-16R-LLPS is observed throughout the neurons but does not form punctate condensates (Fig. 2B). Collectively, taken in conjunction with the previously published validation of these mutations, this data confirms the specificity of the mutations introduced into FUS-16R.

### FUS-16R condensates impaired PSD-95 expression in a manner dependent upon dendritic localisation and abnormal condensation of FUS

FUS condensates induced by fALS-FUS-associated mutations lead to synapse weakening and ultimately cause synaptic dysfunction [4, 14, 16]. To determine whether the FUS-16R mimic of hypomethylated FUS might have the same effect in CA1 hippocampal neurons, we next examined the expression of postsynaptic density 95 (PSD-95)—a key marker of excitatory synapses. We coexpressed either the wild-type FUS or FUS-16R constructs (described above) together with a fibronectin intrabody generated with mRNA display (FingRs), which binds and tags endogenous PSD-95 with GFP [25] (Fig. 2C). We found that both the density and the area of PSD-95 puncta were significantly reduced in neurons expressing FUS-16R condensates compared to control neurons (expressing td-Tomato) and neurons expressing FUS-WT (*density*: Fig. 2D; FUS-16R vs control p < 0.0001; FUS-16R vs FUS-WT, p < 0.0001, post hoc Tukey; *area*: Fig. 2E; FUS-16R vs control, p < 0.045; FUS-16R vs FUS-WT, p < 0.0058, post hoc Tukey).

Next, to determine whether this reduction in PSD-95 puncta was driven primarily by aberrant localisation or by aberrant fibrillar condensation of the FUS-16R protein, we repeated these experiments using either the FUS-16R-NLS [20] or the FUS-16R LLPS [19] constructs described above (Fig. 2C). We found that both displacement of the FUS-16R condensates into the nucleus (by FUS-16R-NLS), or abrogation of the ability of FUS-16R to assemble into stable fibrillar condensates (by FUS-16R-LLPS) significantly rescued PSD-95 puncta density and puncta area (*density*: Fig. 2D; FUS-16R vs FUS-16R-NLS, p < 0.0001; FUS-16R vs FUS-16R-LLPS. p < 0.0001, post hoc Tukey; *area*: Fig. 2E; FUS-16R vs FUS-16R-NLS, p < 0.0118; FUS-16R vs FUS-16R-LLPS, p < 0.0229, post hoc Tukey). These experiments reveal that both mis-localisation and aberrant fibrillar condensation are necessary factors in disrupting physiological synaptic architecture by the FUS-16R hypomethylation mimic.

### FUS-16R condensates impair AMPA- and NMDA-receptor function in a manner dependent upon dendritic localisation and aberrant condensation of FUS

**Fig. 3:**
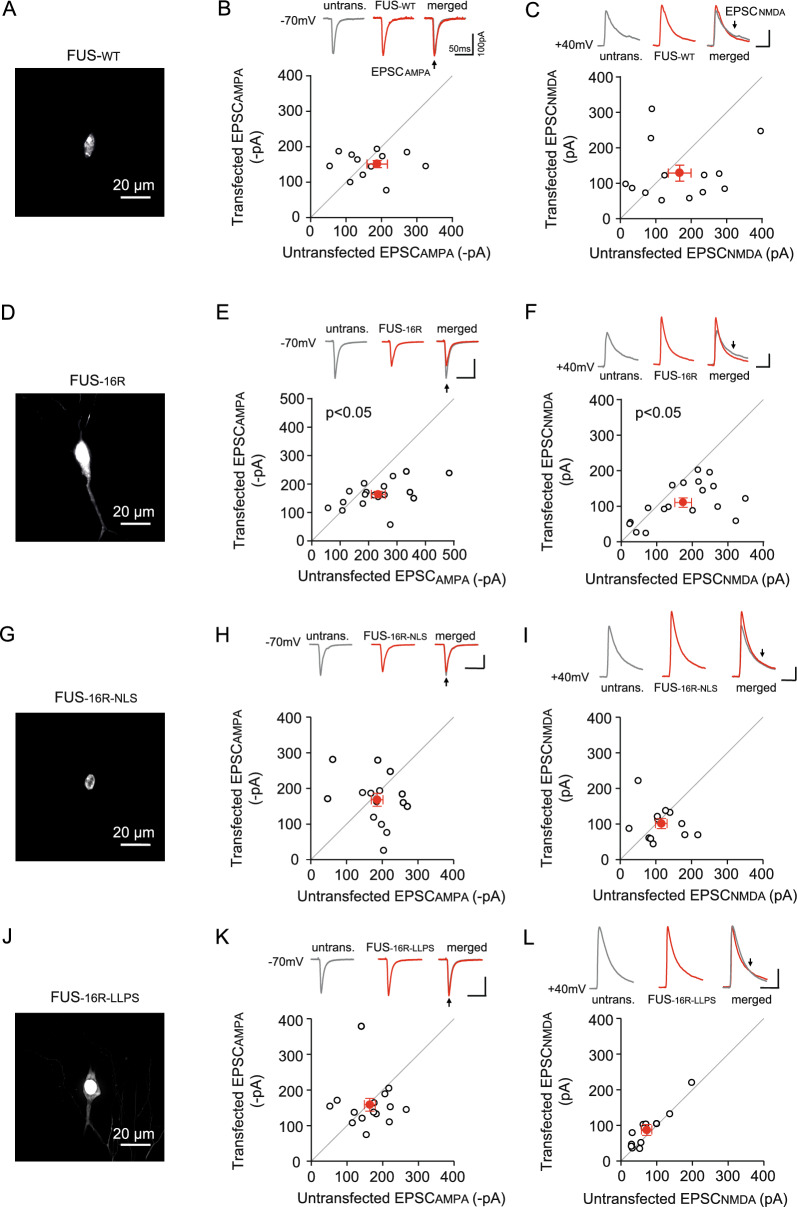
FUS-16R induced a reduction of AMPA- and NMDA-R evoked EPSCs in a manner which is dependent on its localisation and ability to undergo liquid–liquid phase separation*.* **A**, **D**, **G**, **J** Representative widefield images illustrating the FUS expression and localisation for FUS-WT (**A**), FUS-16R (**D**), FUS-16R-NLS (**G**) and FUS-16R-LLPS (**J**) transfected neurons. **B**, **C**, **E**, **F**, **H**, **I**, **K**, **L** Pair-wise analysis of the amplitude of basal EPSCs (EPSC_AMPA_ and EPSC_NMDA_) between untransfected and neighbouring transfected neurons for FUS-WT (**B**, **C**), FUS-16R (**E**, **F**), FUS-16R-NLS (**H**, **I**) and FUS-16R-LLPS (**K**, **L**). Representative single trances of EPSC_AMPA_ and EPSC_NMDA_ recorded at − 70 and + 40 mV holding current respectively, and evoked via Schaffer Collateral stimulation, black arrow indicates peak amplitude measurement. P < 0.05, unpaired t-test

The abnormal dendritic localisation and hypermotility of the FUS-16R condensates prompted us to examine their effect on synaptic function. Focusing on post-synaptic CA1 neurons of the Schaffer Collateral synapse, we evoked AMPA and NMDA receptor-mediated excitatory post-synaptic currents (EPSC) by electrically stimulated the Schaffer Collateral afferents and recording the evoked EPSC simultaneously from a transfected and untransfected neighbouring neuron (Fig. 3A-L). Pair-wise analysis of EPSCs indicated that expression of FUS-WT did not induce any deficit in EPSC_AMPA_ and EPSC_NMDA_ (Fig. 3A-C; EPSC_AMPA_ p = 0.4728, n = 13; EPSC_NMDA_ p = 0.3273, n = 13; unpaired t-test). However, both the EPSC_AMPA_ and EPSC_NMDA_ were decreased in FUS-16R transfected neurons compared to untransfected neurons (Fig. 3D-F; EPSC_AMPA_ p = 0.014, n = 18; EPSC_NMDA_ p = 0.024, n = 18; unpaired t-test). Crucially, however, these electrophysiological deficits are rescued by both FUS-16R-NLS and by FUS-16R-LLPS (*FUS-16R-NLS*: Fig. 3G-I; EPSC_AMPA_ p = 0.484, n = 15*;* EPSC_NMDA_ p = 0.531, n = 12, unpaired t-test; *FUS-16R-LLPS*: Fig. 3J-L; EPSC_AMPA_ p = 0.362, n = 15; EPSC_NMDA_ p = 0.312, n = 12, Mann–Whitney test). Thus, the FUS-16R can induce impairments in basal synaptic function, specifically AMPAR and NMDAR mediated EPSCs, which arise from the dendritic mis-localisation and aberrant condensation of the FUS-16R hypomethylation mimic.

### FUS-16R condensates impaired single spine plasticity in a manner dependent upon dendritic FUS localisation and pathological condensation

**Fig. 4:**
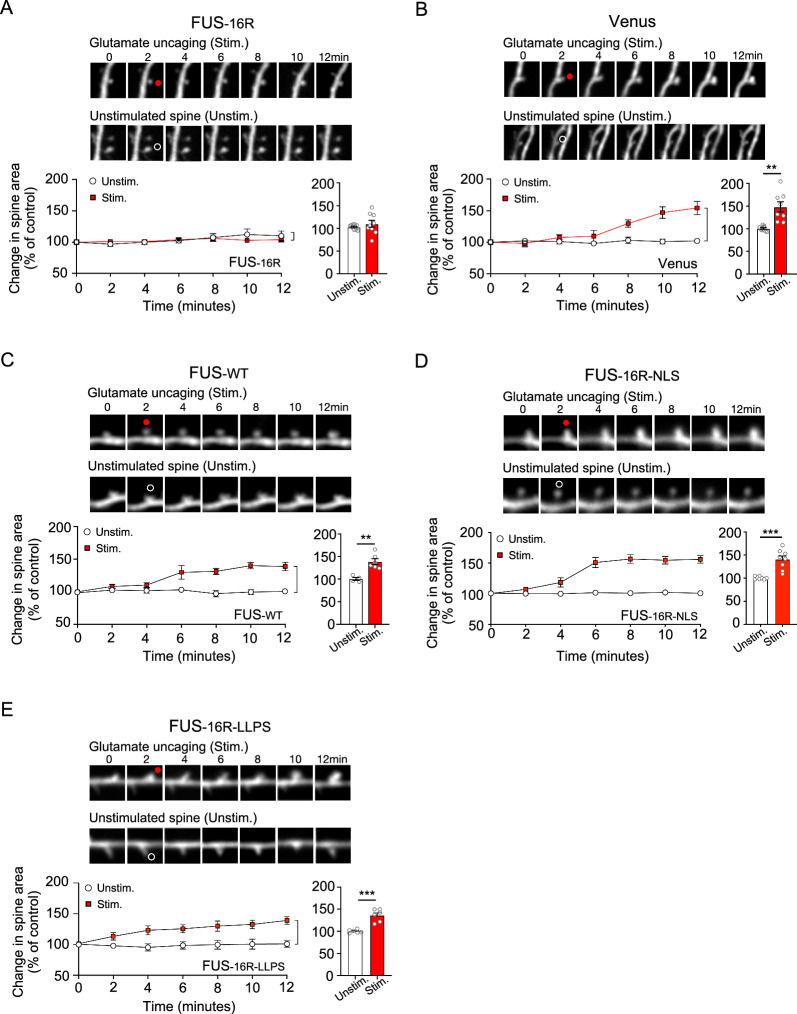
FUS-16R induced synapse dysfunction, inhibiting the initiation of single spine structural plasticity*.* **A**–**E** Representative time course images of dendritic spines prior to and following single spine glutamate uncaging for stimulated spines (top) and unstimulated spines (bottom) for neurons transfected with FUS-16R (**A**), Venus (structural marker control) (**B**), FUS-WT (**C**), and FUS-16R-NLS (**D**) and FUS-16R-LLPS (**E**). The corresponding time course graph illustrating the average change in spine head areas, as a percentage of baseline, and histogram illustrating the final (12-min post stimulation) change in area for FUS-16R (a; p = 0.405, n = 9), Venus (b**;** p = 0.003, n = 8), FUS-WT (c; p = 0.008, n = 6,), and FUS-16R-NLS (d**;** p = 0.001, n = 8,) and FUS-16R-LLPS (e; p = .0001, n = 6). ** p < 0.01, ***p < 0.001, unpaired t-test

We next wished to discover whether the observed changes in synaptic protein dynamics might affect the induction of activity-dependent dendritic spine plasticity in CA1 neurons. Activity-dependent spine plasticity is associated with both functional and structural modification of dendritic spines [26], and underlies the cellular and molecular mechanism of learning and memory [26, 27]. To accomplish this, we measured changes in single spine head size before and after the uncaging of glutamate (MNI-Glutamate) at apical dendritic spines (Fig. 4).

To monitor spine structure as a marker of synaptic plasticity, we transfected control neurons, FUS-WT neurons, FUS-16R neurons, FUS-16R-NLS and FUS-16R-LLPS neurons with Venus, a fluorescent protein frequently utilised to monitor spine structure. Venus-transfected control neurons and Venus-transfected FUS-WT neurons both demonstrated physiological increases in spine size in response to glutamate uncaging (*Control*: p = 0.003, unpaired t-test, n = 8, Fig. 4B; *FUS-WT:* p = 0.008, unpaired t-test, n = 6, Fig. 4C). In contrast, spine size did not change in FUS-16R-expressing neurons (p = 0.405, unpaired t-test, n = 9, Fig. 4A). This loss of spine plasticity was rescued in neurons expressing either FUS-16R-NLS or FUS-16R-LLPS. Thus, neurons expressing either FUS-16R-NLS or FUS-16R-LLPS exhibited plasticity levels similar to control and FUS-WT expressing neurons (*FUS-16R-NLS*: p = 0.001, unpaired t-test, n = 8, Fig. 4D; *FUS-16R-LLPS:* p = 0.0001, unpaired t-test, n = 6, Fig. 4E). Collectively, this data reveals that both dendritic mis-localisation and aberrant condensation of FUS-16R very significantly disrupt the initiation of synaptic plasticity. And they likely do so by disrupting the dynamic, activity -dependent changes in the expression of key proteins within the postsynaptic dendrite and dendritic spine.

### The presence of dendritic FUS-16R condensates can impair the fluorescent recovery of photobleached PSD-95

**Fig. 5:**
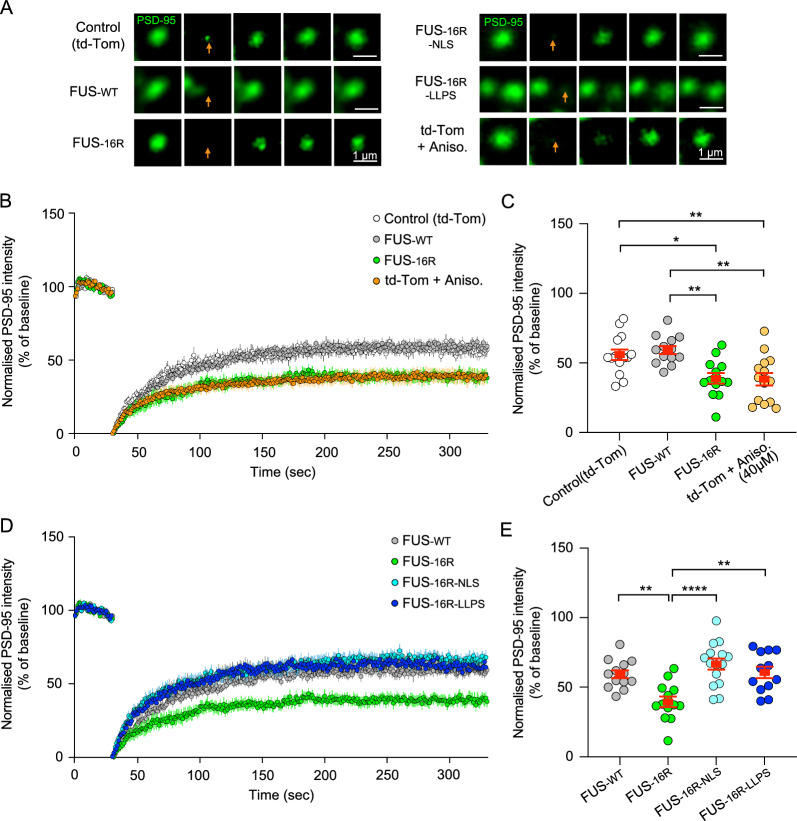
FUS-16R impaired the homeostatic movement of PSD-95 within the synapse. **A** Representative time course images of endogenous PSD95 puncta prior to and post photobleaching (arrow) for Control, FUS-WT, FUS-16R, FUS-16R-NLS, FUS-16R-LLPS transfected neurons and control neurons preincubated in anisomycin. **B**, **C** Average normalised fluorescence intensity recovery of the photobleached endogenous PSD95 puncta over time and at 5 s prior to the final time point for Control (white circles; n = 14), FUS-WT (grey circles; n = 13), FUS-16R (green circles; n = 13) transfected neurons and Anisomycin-treated neurons (orange circle; n = 14). **D**, **E** Average normalised fluorescence intensity recovery of the photobleached endogenous PSD95 puncta over time and at 5 s prior to the final time point for FUS-WT (grey circles; n = 13), FUS-16R (green circles; n = 13), FUS-16R-NLS (cyan circles; n = 15) and FUS-16R-LLPS (blue circles; n = 12) (**F**). *p < 0.05, ** p < 0.01, **** p < 0.0001, post hoc Tukey analysis

FUS binds RNA and plays a key role in RNA transport and translation [3, 28]. FUS also interacts with key synaptic proteins such as PSD-95 [14]. Consequently, we wished to determine whether the abnormal localisation and condensation of FUS-16R in synapses might cause changes in the dynamic expression of synaptic proteins, which could underpin the observed functional impairments. In this experiment, we used PSD-95 as a representative exemplar. We applied a fluorescence recovery after photobleaching (FRAP) approach. We found that the FRAP recovery of PSD-95 was significantly reduced in neurons expressing FUS-16R when compared with control neurons (expressing td-Tomato) and FUS-WT neurons (Fig. 5A-C). Interestingly, in control (TdTomato transfected) neurons, inhibition of new protein translation, and therefore translation of PSD-95, via preincubation with anisomycin (40 μM; 30 min) reduced the PSD-95 FRAP recovery to a similar extent as in FUS-16R neurons (p = 0.9996, post hoc Tukey). These findings suggest that FUS16R condensates impair the normal homeostatic regulation of PSD-95. This could be occurring due to a direct interaction of the FUS-16R condensates with PSD-95 protein or from a sequestering of PSD-95 RNA within the condensates. Further investigation will be required to resolve this question. Finally, we determined that the reduction in PSD-95 FRAP recovery was fully rescued by FUS-16R-NLS and FUS-16R-LLPS (*FUS-16R vs FUS-16R-NLS*, p < 0.0001, post hoc Tukey; *FUS-16R vs FUS-16R-LLPS*, p = 0.0013, post hoc Tukey). Indeed, both FUS-16R-NLS and FUS-16R-LLPS exhibited similar levels of PSD-95 FRAP recovery to each other (Fig. 5D, E) and to control and FUS-WT neurons (Fig. 5D, E). Collectively, these experiments again support the hypothesis that both the dendritic mis-localisation of FUS-16R and its propensity to form stable fibrillar condensates significantly disrupt the normal activity-dependent changes in the dynamic expression of at least some key synaptic proteins (i.e., PSD-95).

## Discussion

Prior work examining the pathobiology of FUS condensates has focused on condensates induced by fALS-FUS mutations. Three hypotheses have been proposed: 1) Nuclear loss of function (e.g., impairment of transcription), 2) Loss of the normal cytoplasmic role of FUS, 3) Toxic gain of function (e.g., sequestration of RNA/proteins inside condensates) [14, 29–31]. These hypotheses are not mutually exclusive. However, the latter hypothesis is supported by several studies [4, 32–34], some of which show that driving the fibrillar stable condensates from the cytoplasm into the nucleus reduces synapto-toxicity [35–37].

In contrast, the mechanisms by which arginine hypomethylated FUS condensates cause neuronal dysfunction in sporadic FTLD-FUS is still poorly understood. A major impediment to the field has been the lack of suitable tools. This obstacle arises from the absence of knowledge about the enzymatic processes that promote the accumulation of hypomethylated FUS. As a result, until recently, the only method for investigating the neurobiological impact of arginine hypomethylated FUS condensates has been through the use of small molecule inhibitors of one carbon metabolism (e.g. AdOx). However, these compounds have broad effects on the methylation states of both DNA and multiple other proteins, thereby adding many experimental confounds.

Fortunately, recent work by this and other groups have generated powerful new insights into the biophysical mechanism driving the propensity of arginine hypomethylated FUS to form stable fibrillar condensates [2, 17]. This work reveals that the propensity of hypomethylated FUS to over-condensation into stable fibrillar assemblies arises from the increased interaction of protons in the demethylated arginine guanidino moiety with pi electrons in aromatic ring of tyrosines. We previously took advantage of this biophysical insight to generate a series of FUS protein constructs (epitomised here by FUS-16R) that incorporate additional arginine residues [2]. These additional arginine residues increase the cation-pi drive and thereby increase the propensity of hypomethylated FUS to condense into ensembles that mimic the biophysical properties of demethylated FUS. Crucially, the CD spectra of FUS-16R, wild type physiologically methylated FUS and wild-type hypomethylated FUS are indistinguishable [2]. This indicates that the increased condensation propensity of FUS-16R is not due to simple misfolding and aggregation [2].

Using this molecular tool, we now demonstrate that FUS-16R causes: aberrant accumulation of pathological FUS-16R condensates in dendrites and dendritic spines which exhibit both baseline and activity-dependent hypermotility, and these pathological condensates can be recruited into dendritic spines; impaired dynamic expression of a key post synaptic protein—PSD95; impaired postsynaptic AMPA and NMDA receptor-mediated excitatory postsynaptic currents; and impaired synaptic remodelling, with the failure to enlarge synaptic spines following glutamate activation.

We show that these synaptic effects are dependent upon both the formation and dendritic mis-localisation of FUS-16R condensates. Crucially, these synaptic effects are rescued by forced re-localisation of the FUS-16R into the nucleus (using FUS-16R-NLS). The synaptic effects can also be rescued by reducing the number of tyrosine residues and thus attenuating the cation-pi-mediated condensation of FUS-16R (using FUS-16R-LLPS). We also illustrated that FUS-16R forms more condensed assemblies (most likely gel and/or fibrillar aggregates). As such, we can state that FUS-16R undergoes LLPS and then progresses to form hyper-condensed assemblies. It is then these hyper condensed assemblies located in the dendritic regions which result in synapse weakening. These rescue effects are fully congruent with rescue effects observed in similar experiments in neurons expressing fALS-FUS mutants [32]. However, as the FUS-16R condensates are widespread it is possible that they could be impairing other key machinery within the neurons (e.g. transport, mitochondria etc.) which could have downstream effects on synaptic function. However, this is out width the scope of the current study and would require further investigation.

We show that one key downstream effect of pathological FUS-16R condensates is to alter the dynamic expression of key synaptic proteins such as PSD-95. PSD-95 is highly expressed in the postsynaptic compartment and plays an important role in surface presentation of AMPA receptors and in synaptic structure [36, 37, 41]. Both PSD-95 and AMPA receptors are critical for the long-term synaptic plasticity [38–40]. FUS appears to be intimately linked with these processes. Thus, FUS interacts with the mRNAs for key synaptic proteins (including GluA1 mRNA [15]). FUS also directly interacts with some of these key synaptic proteins, including AMPA, NMDA and CaMKII [14]. We hypothesise that mis-localisation and abnormal condensation of FUS-16R mimic of arginine hypomethylated FUS could disrupt synaptic function by altering the localisation and/or availability of these key synaptic proteins and their cognate mRNAs.

The work reported here directly supports these hypotheses. Thus, we show that FUS-16R condensates, which mimic pathological hypomethylated FUS condensates, influence the dynamics of FUS condensates in dendrites and dendritic spines. We also show that they affect the expression of PSD-95 protein. These protein expression-based changes are coupled with electrophysiological and synaptic morphology changes that are likely downstream consequences of these protein changes.

Collectively, our findings illustrate that FUS-16R condensates can impair the homeostatic function of PSD-95. However, our work does not presently identify the precise molecular mechanism by which aberrantly stable FUS-16R condensates impact the expression of PSD-95 and other synaptic proteins. However, we can propose that for PSD-95, there are at least two potential mechanisms by which pathologically condensed FUS-16R RNP granules in dendrites and dendritic spines could disrupt RNA processing for synaptic proteins like PSD-95. Firstly, the presence of the FUS-16R condensates in the dendrites and spines could impair the transport of key proteins such as PSD-95. Secondly FUS is known to bind mRNAs for synaptic RAS GTPase-activating protein 1 (SynGAP), which is essential for maintaining and stabilising the synaptic/surface expression of PSD-95 [41]. Alternatively, prior work has shown that dendritic localisation of PSD-95 mRNA directly regulates PSD95 translation [42]. Consequently, PSD-95 protein expression could be attenuated by mis-localisation and/or sequestration of these key mRNA’s within pathological FUS-16R condensates. While this discussion focuses on PSD-95, we anticipate that other key synaptic components might be similarly influenced, and thereby contribute to the synaptic dysfunction. Appropriate additional experiments can be envisaged to address these unanswered questions.

In addition, the role of FUS and FUS mutations is relatively well documented in the presynaptic compartment [14, 34, 43]. However, there is limited research on the impact of FUS hypomethylation at either the pre- or post-synapse. Our study utilised the organotypic hippocampal slice culture model coupled with biolistic transfection, optimised to observe a low transfection ratio into the CA1 hippocampal neuron, where there is a well characterised pre-post synaptic circuit in which the postsynaptic dendritic spine plays an important role in molecular mechanism of learning and memory. This experimental design raises the likelihood that the findings presented in this study arise from the post synaptic expression of FUS-16R therefore we eliminated possible presynaptic disturbances (i.e., only the postsynaptic neuron expressed FUS-16R). However, based on the known roles of FUS at the presynapse, further investigation into the potential pathophysiological role of hypomethylated FUS condensates at the presynaptic compartment would be beneficial.

We observed that FUS-16R condensates exhibited both spontaneous and activity induced movement within the dendritic regions of the CA1 neurons. The molecular basis of this finding is not immediately apparent. RNP granules typically do not have motor protein attachments. However, we have shown that RNP granules can be tethered to the surface of lysosomes via annexin 11 and then hitchhike on classical intracellular motors [44]. Future work will be needed to discern whether the hypermobility reflects persistent links of pathological FUS-16R condensates to these RNP granule transport systems, and if the activity dependent recruitment of FUS-16R condensates to dendritic spines directly impairs synaptic function. If so, this might further contribute to impaired regulation of local new protein synthesis in synaptic terminals. Furthermore, the conditional knockout of FUS in the hippocampus was shown to alter excitatory synaptic function and lead to behavioural disinhibition [13], a hallmark of FTLD, therefore, in our model, the loss of synapse function by hypomethylation mimic of FUS could explain the underlying pathophysiology which drives behavioural changes.

Here we present data which illustrates a pathophysiogical role of hypomethylated FUS in the hippocampus, this is in keeping with research illustrating FUS condensates are observed in patient post-mortem hippocampal tissue [12] and the known role for FUS in mediating excitatory transmission [13, 15]. Specifically, a global hippocampal knock down of FUS was shown to reduce excitatory postsynaptic currents (EPSCs) and reduce mature (mushroom) spine structures in a manner dependent on GluA1 [15]. Similarly, we observe a reduction in AMPAR and NMDAR mediated EPSC and observed a loss of spines, potentially indicating that FUS16R (i.e., hypomethylated FUS) induced a greater synaptic dysfunction. However, a recent study illustrated different roles for FUS in specific hippocampal compartments showing that regional knock down of FUS induced decreased excitatory transmission in the intermediate hippocampus and increased excitatory transmission in the ventral hippocampus [13]. As we did not design the current study to examine different hippocampal subregions we cannot rule out the region specific pathophysiology.

Recent studies have shown wild type FUS RNP granules can be located at synaptic compartments, however many of these studies have utilised super-resolution microscopy or subcellular fractionation [14, 43, 45]. Thereby suggesting that the level of FUS located at the synaptic compartments in ‘healthy’ neurons is small in comparison to its abundant expression in the nucleus. Furthermore, the accumulation of wild type FUS at synaptic compartments has been associated with a pathological phenotype of neurodegeneration [46]. Therefore, it is not surprising that we were unable to observe wild type FUS at synaptic compartments during live cell imaging of organotypic slice culture.

There remain several important questions surrounding the neurobiology of pathological FUS condensates (either from fALS-FUS mutations or FTLD-FUS arginine hypomethylation). For instance, FUS is widely expressed in many cell types. Why then are FUSopathies predominantly manifest by neurodegeneration? Why does arginine hypomethylation of FUS predominantly target frontotemporal cortical neurons whereas the fALS-FUS mutants predominantly target upper and lower motor neurons?

An early hypothesis regarding the former question was that, compared to smaller cell types, the extremely elongated corticospinal and spinal motor neurons might be more sensitive to the impaired transport of pathologically condensed fibrillar FUS RNP granules. However, while attractive, this hypothesis does not explain the susceptibility of temporal and hippocampal neurons (with shorter axons). The hypothesis also does not explain the relative resilience of equally long ascending sensory neurons.

However, there is an alternate explanation for the phenotypic differences associated with the pathological condensates induced via arginine hypomethylation (FTLD-FUS) versus those associated with missense mutations (fALS-FUS). The difference in the formation of the condensates (e.g., hypomethylated vs methylated) may result in subtle differences in the RNA and protein interactomes of wild type FUS, missense mutant FUS, and hypomethylated FUS. Further supporting this hypothesis is the fact we observe FUS-16R to form more condensed gel and/or fibrillar aggregates, whereas the ALS associated mutation FUSP525L condensates displayed liquid-like properties [20]. It is conceivable that the differing ALS versus FTLD phenotypes reflect the impact of hypomethylated arginine residues or of missense mutation on different, cell-type specific cargo elements that that are misprocessed by the pathological, stable fibrillar FUS condensates, however these hypothesis require further investigation.

## Conclusion

Mimicking the pathological hypomethylation of FUS (e.g., FUS-16R) induced dendritic condensates and impaired synaptic function. Crucially, we have identified that both the dendritic localisation of the condensates, and their ability to undergo LLPS and form stable condensates is essential for driving synapse weakening. These results highlight the importance of the formation and localisation of the FUS condensates as a key component in hypomethylated FUS pathophysiology. Interestingly. FUS hypomethylated condensates have been observed in sporadic FTD cases. Clearly, these important questions will require additional work. Nevertheless, the experiments described here may have practical applications. Specifically, these experiments provide a potential platform through which to screen and preclinically validate compounds that can could be used to therapeutically manipulate abnormal dendritic FUS-16R phase state (e.g., PhaseScan technology) [47–49]. If successful, such compounds could potentially be used in the symptomatic management of patients with FTLD-FUS even in the absence of an understanding of the enzymology of arginine hypomethylation in FUS.

## Materials and methods

### Animals

All procedures involving animals were carried out in accordance with the UK Animals Scientific Procedures Act, 1986. Male 7-day old Wistar rats (Charles River, UK) were used to prepare organotypic hippocampal slices. All animal experiments were given ethical approval by the ethics committee of University of Bristol or King’s College London (protocol reference U214) (United Kingdom).

### Organotypic Hippocampal Slice Culture Preparation

Organotypic slices were cultured as previously described [50]. All steps were carried out under sterile conditions. Briefly, rats were decapitated, and their brains rapidly removed and placed into ice-cold dissecting medium containing: sucrose (238 mM), KCl (2.5 mM), NaHCO_3_ (26 mM), NaH_2_PO_4_ (1 mM), MgCl_2_ (5 mM), D-glucose (11 mM) and CaCl_2_ (1 mM). Hippocampi were extracted and transverse hippocampal slices were cut and placed upon sterile, semi-porous membranes (Merck Millipore, USA) and stored at the interface between air and culture medium containing: 78.8% minimum essential medium with L-glutamine, 20% heat-inactivated horse serum, HEPES (30 mM), D-glucose (26 mM), NaHCO_3_ (5.8 mM), CaCl_2_ (2 mM), MgSO_4_ (2 mM), ascorbic acid (70 μM) and insulin (1 μg ml^−1^) (pH adjusted to 7.3 and 320—330 mOsm kg^−1^), inside a humidified incubator at 35 °C with a 5% CO_2_ enriched atmosphere.

### Biolistic Transfection and Plasmid information

Organotypic slices were biolistically transfected at DIV 4–5 using the Helios Gene Gun system (Bio-Rad, USA). The FUS-16R mutant [G167R, G170R, G173R, G202R, S205R, S221R, G225R, G228R, G230R, M254R, G379R, N381G, insert387G, G398R, G401R, S402G, G404R, G456R, M464R] was described previously [2]. The Tyrosine to Serine substitutions to render FUS LLPS-deficient [Y6S, Y14S, Y17S, Y25S, Y33S, Y37S, Y41S, Y50S, Y55S, Y58S, Y66S, Y75S, Y81S, Y91S, Y97S, Y100S, Y113S, Y122S, Y130S, Y136S, Y143S, Y150S, Y155S, Y161S, Y177S, Y194S, Y208S] were described previously [19]. Constructs with full-length (1–526) wildtype FUS and the 16R peYFP-C1 vector (Clontech). pcDNA6-TdTomato-FUS-16R-NLS and pcDNA6-TdTomato-FUS-16R-LLPS were generated as follows: The optimised coding sequences of 16R-FUS either fused to a c-terminal SV40 NLS (5’- CCTAAGAAGAAGCGGAAGGTCGAGGACAAG-3’) [20] or containing 27 tyrosine to serine substitutions in the N-terminal domain (according to [19] and [20]) were ordered by Gene synthesis (GeneArt, Life Technologies) and cloned into XhoI-NotI sites of pcDNA6-EGFP-GSG15 (described in Reber et al. [20]). EGFP was then exchanged with TdTomato by cloning an AgeI-SalI digested TdTomato PCR fragment in AgeI-SalI digested pcDNA6-EGFP-GSG15-FUS-16R-NLS and pcDNA6-EGFP-GSG15-FUS-16R-LLPS, respectively. TdTomato cDNA was amplified from TdTomato-C1 (a gift from Michael Davidson, Addgene plasmid # 54,653; http://n2t.net/addgene:54653; RRID:Addgene_54653) using Platinum Superfi II MasterMix according to the manufacturer’s instructions with primers mdr5 (5’-TTGACGCAAATGGGCGGTAG-3’) and DJ287 (5’- CCTCTACAAATGTGGTATGG-3’). Constructs were verified by Sanger Sequencing. Endogenous PDS-95 was labelled in “live” neurons by using a PSD95.FingR-GFP plasmid which binds to endogenous PSD-95 upon which excess unbound PSD95.FingR-GFP returns the nucleus and exhibits transcription control resulting in PSD95.FingR-GFP expression which mirrors the expression of endogenous PSD-95 (as described Gross et al. [25]). The red-shifted channel rhodopsin C1Chrimson(S169A) was kindly provided by Prof Nureki and Dr Oda [24]. When appropriate fluorescent volume filling constructs were utilised to visualise spine morphology and area, here we utilised either TdTomato or Venus, as described in the text.

### Electrophysiological Recordings

Whole-cell electrophysiology recordings were made from CA1 neurons of hippocampal organotypic slice cultures at DIV 9–12 and DAT 5–7. The slice cultures were maintained in the recording chamber perfused with a buffer solution containing NaCl (119 mM), KCl (2.5 mM), CaCl_2_ (4 mM), MgCl_2_ (4 mM), NaHCO_3_ (26 mM), NaH_2_PO_4_ (1 mM), glucose (11 mM), picrotoxin (0.04 mM), and 2-chloroadenosine (0.01 mM), at 29–30 °C, saturated with 95% O_2_/5% CO_2_. A bipolar stimulating electrode was placed on the Schaffer collateral pathway and patch electrodes (5–6 MΩ) containing CsMeSO_4_ filling solution (comprising CsMeSO_4_ (130 mM), NaCl (8 mM), Mg-ATP (4 mM), Na-GTP (0.3 mM), EGTA (0.5 mM), HEPES (10 mM), and QX-314 (6 mM), pH 7.2–7.3 and 270–290 mOsm/kg) were used to patch and voltage clamp CA1 pyramidal neurons. Only cells with an initial Rs < 20 MΩ that was maintained at a level within 20% of that value until experiment completion were included for data analysis. EPSC_AMPA_ was measured as the peak EPSC amplitude at the holding potential of -70 mV and EPSC_NMDA_ was measured as the peak EPSC amplitude 90–100 ms after stimulus at a holding potential of + 40 mV. For each neuronal pair 10 EPSC_AMPA/NMDA_ responses were recorded and averaged. Data were filtered at 2 kHz and digitized at 20 kHz using a Multiclamp 700A amplifier. Data were recorded and online data analysis was performed using WinLTP software (WinLTP Ltd, UK). Offline data analysis was performed with WinLTP.

### Glutamate uncaging assays

Organotypic slices were submerged in a low Mg^2+^ HEPES buffer and images acquired at room temperature on a multiphoton system (Scientifica Hyperscope with a Coherent Chameleon Discovery; Nikon 16x, 0.8 NA lens or a Nikon 25x, 1.1 NA lens). A region of interest containing dendritic spines located on apical secondary dendritic branches (100–200 μM from Soma). A small z-stack was obtained (0.5 μM step size). This initial image was used to identify the target spine. For induction of plasticity assays, a time-lapse acquisition was acquired the same z-stack every 2 min. Two-photon stimulation (2 Hz, 100 pulses, 10 ms, 5 mW, 720 nm) was aimed at the tip of the spines to uncage 5 mM MNI-glutamate (HelloBio, UK). Spine head area was calculated by summating the z-stack and processing via Image-J. For glutamate uncaging induced FUS16R granule movement assays, a time-lapse acquisition acquired the same z-stack every 10 min. Following a base line period glutamate was uncaged (as described above) and timelapse z-stack images acquired at 10 min intervals for a further 30 min. Change in FUS-16R was calculated from a change in the fluorescent intensity at each interval by summating the z-stack and processing via Image-J and performing a line-scan across the spine head.

### Fluorescence recovery after photobleaching (FRAP) assay

PSD-95 puncta were located at regions of interest on apical secondary dendritic branches. A time-lapse acquisition (16x, 0.8 NA lens) acquired at 2 Hz for 30 s as a baseline. Photobleaching (2 Hz, 40 pulses, 300 ms, 35 mW, 920 nm, 0.45 µm × 0.45 µm log spiral shape) aimed on individual PSD-95 puncta. Immediately following photobleaching a further time-lapse acquisition was obtained at 2 Hz for 5 min. Offline, a group average was applied to average every 2 images from time-lapse and fluorescence intensity was measured at the target PSD-95 puncta via Image-J. The fluorescence intensity values were normalised to the baseline intensity.

### Spinning Disk Confocal imaging

Transfected neurons were imaged at DIV 9–12 and DAT 5–7. Slices were submerged in a HEPES buffer and images acquired using Nikon-Yokogawa Spinning Disk confocal microscope with Nikon 100 × 1.10 NA lens.

### PSD-95 puncta analysis

Organotypic slices were submerged in a HEPES buffer and images acquired at room temperature on a custom spinning disk confocal microscope (Nikon, Japan) with laser point stimulation (Rapp-Opto, Germany). A z-stack of a region of interest containing a secondary apical dendritic branch was acquired (8 averages per Z-frame). From the image a smaller ROI (20 μM) was selected and the z-stack averaged. The ROI was post processed in Fiji (imageJ) to reduce background noise. For PSD-95 puncta analysis the image was thresholded, and the thresholded puncta automatically analysed for area and number.

### Spontaneous FUS-16R granule movement assay

The spinning disk confocal microscope was utilised to identify a dendritic region of interest, as described above. A single z-plane image was acquired for 1 min at 2 Hz capturing the localisation of the FUS-16R condensates. Offline analysis was performed with ImageJ plugin, Mosaic Suit. FUS-16R granules 5 pixels or bigger were tracked and only trajectories which persisted for 5 frames were further analysed. Total granule trajectory length (μm/min) and average granule movement (μm/sec) were tracked and calculated.

#### Chrimson induced FUS-16R granule movement assay

A secondary apical dendritic branch was identified, and an image acquired on the spinning disk confocal microscope. A single z-plane image was acquired for 1 min at 2 Hz to capturing the localisation of the FUS-16R condensates. Immediately following the acquisition Chrimson was activated at the dendritic region of interest (200 ms, 0.4 Hz, 5 min, with a 647 nm; Rapp-Opto laser stimulation module) following which a further min was acquired. Analysis was performed as described above for the spontaneous granule movement assay.

#### Validation of FUS constructs and 1,6 Hexanediol in HEK293T

HEK293T cells were cultured in DMEM-High glucose, pyruvate, not glutamate plus 10% foetal bovine serum, 1 × GlutaMax and 1 × Antibiotic–Antimycotic. Once 80% confluent, cells were transfected with the various FUS constructs (1 μg/μL) by lipofectamine 2000 (Thermofisher Scientific, UK) following manufacture guidelines. Subsequently, 24 h after transfection images were acquired using a multiphoton system (Scientifica Hyperscope with a Coherent Chameleon Discovery; a Nikon 25x, 1.1 NA lens). Average z-projections were created using FIJI (ImageJ). HEK293T cells transfected with FUS-16R were imaged at room temperature on a multiphoton system (Scientifica Hyperscope with a Coherent Chameleon Discovery; Nikon 16x, 0.8 NA lens or a Nikon 25x, 1.1 NA lens). Small Z-Stacks were acquired (0.5 μM step size) following which 10% 1,6 Hexanediol was added to the imaging media. The cells were maintained in the 1,6 Hexanediol media for 45 min and subsequently reimaged. The area of the FUS-16R condensates was calculated using FIJI (Image J), by thresholding and creating a binary image and the analyse particles function to determine the area.

#### Statistics

Statistical analysis was performed using GraphPad Prism 9.0 software. Details of individual statistical tests are provided within Additional file 1: Table S1. For most analyses, paired two-tailed t-tests were performed, with an alpha level of 0.05. For comparisons of effects across multiple groups, one-way ANOVAs were performed followed by post hoc analysis. Statistical analysis of changes in FUS-16R puncta area following addition of 1,6 Hexanediol was calculated by a nested t-test. Sample sizes are described in the relevant sections and were based upon preliminary findings of the minimum number of samples required to detect statistically significant difference in the grouped means, given the observed variance at a power level of 0.8. All grouped data are presented as mean ± SEM.

### Supplementary Information

**Additional file 1: Supplemental Fig. 1.** FUS-16R condensates do not exhibit liquid-like properties. A) Representative image of FUS-16R condensates within HEK293T cells prior to and in the presence (45 minute incubation) of 10% 1,6 Hexanediol. B) Quantification of the area of 90 condensates from 5 HEK293T cells prior to and following incubation with 1,6 Hexanediol. T(8) = 0.0093, P = 0.9255, Nested T-Test. **Supplemental Fig. 2.** Schematic illustration of FUS constructs utilised in the study. Schematic representation of FUS-WT, FUS-16R, FUS-16R-LLPS and FUS-16R-NLS. Mutations associated with creating the hypomethylation mimic (16R) are illustrated in red, impairing liquid-liquid phase separation (LLPS) in green and forcing the nuclear localisation (NLS) in blue.
