# Supplementary material for: Mimicking hypomethylation of FUS requires liquid–liquid phase separation to induce synaptic dysfunctions
Source: Acta Neuropathol Commun. 2023 Dec 18;11:199. doi: 10.1186/s40478-023-01703-w (PMC10726623; doi:10.1186/s40478-023-01703-w)
Supplement: Supplementary file 1 — Additional file 1: Supplemental Fig. 1. FUS-16R condensates do not exhibit liquid-like properties. A) Representative image of FUS-16R condensates within HEK293T cells prior to and in the presence (45 minute incubation) of 10% 1,6 Hexanediol. B) Quantification of the area of 90 condensates from 5 HEK293T cells prior to and following incubation with 1,6 Hexanediol. T(8) = 0.0093, P = 0.9255, Nested T-Test. Supplemental Fig. 2. Schematic illustration of FUS constructs utilised in the study. Schematic representation of FUS-WT, FUS-16R, FUS-16R-LLPS and FUS-16R-NLS. Mutations associated with creating the hypomethylation mimic (16R) are illustrated in red, impairing liquid-liquid phase separation (LLPS) in green and forcing the nuclear localisation (NLS) in blue. [file 40478_2023_1703_MOESM1_ESM.docx]

**
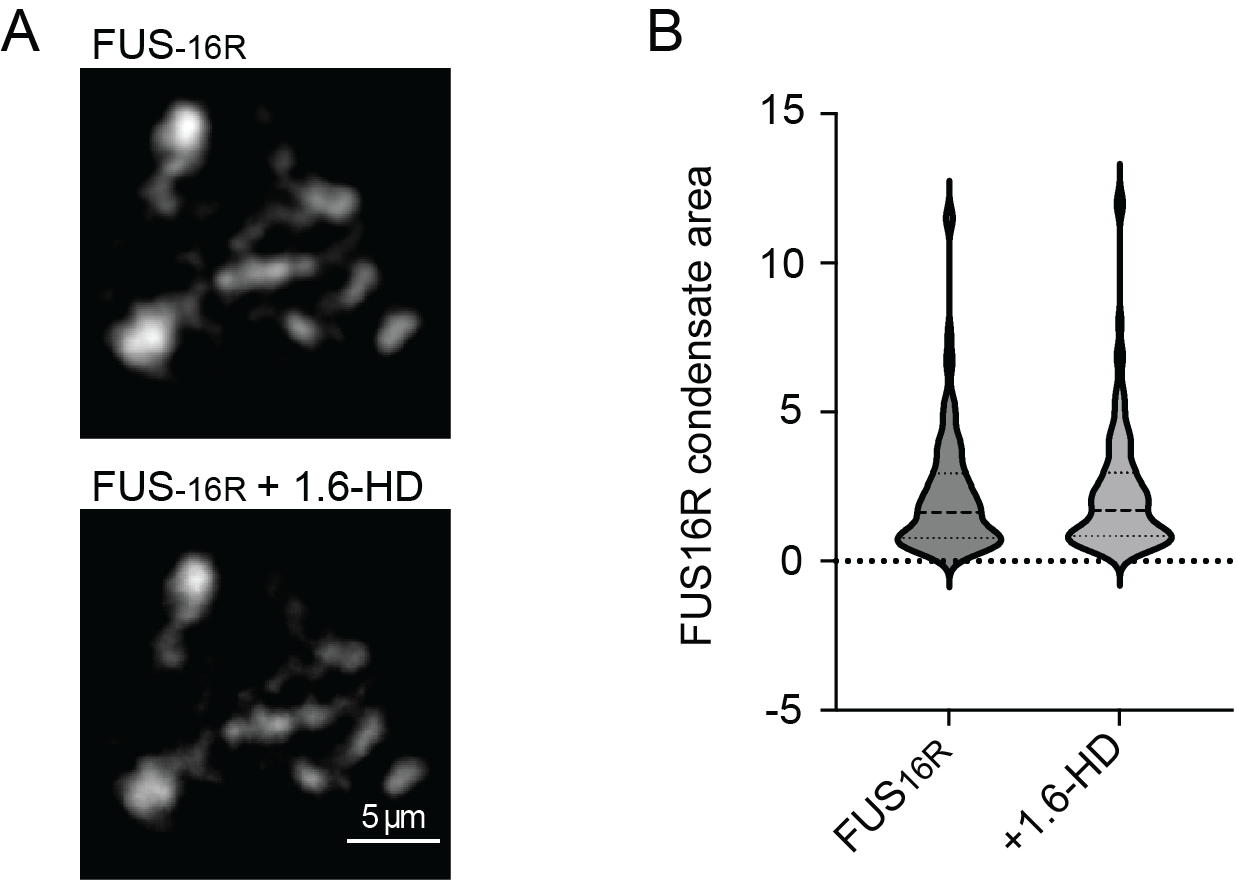
**

**Supplemental Fig. 1. FUS-16R condensates do not exhibit liquid-like properties. A)** Representative image of FUS-16R condensates within HEK293T cells prior to and in the presence (45 minute incubation) of 10% 1,6 Hexanediol. **B)** Quantification of the area of 90 condensates from 5 HEK293T cells prior to and following incubation with 1,6 Hexanediol. T_(8)_ = 0.0093, P = 0.9255, Nested T-Test.

**Supplemental Fig. 2. Schematic illustration of FUS constructs utilised in the study.** Schematic representation of FUS-WT, FUS-16R, FUS-16R-LLPS and FUS-16R-NLS. Mutations associated with creating the hypomethylation mimic (16R) are illustrated in red, impairing liquid-liquid phase separation (LLPS) in green and forcing the nuclear localisation (NLS) in blue.
